# Participatory-informed preference optimization (PiPrO): A reinforcement learning simulation study

**DOI:** 10.1371/journal.pdig.0001294

**Published:** 2026-03-19

**Authors:** Tara Templin, Shuyi Song, Sophia Fort, Nasa Sinnott-Armstrong

**Affiliations:** 1 Department of Health Policy and Management, University of North Carolina at Chapel Hill, Chapel Hill, North Carolina, United States of America; 2 Herbold Computational Biology Program, Fred Hutchinson Cancer Center, Seattle, Washington, United States of America; 3 Cecil G. Sheps Center for Health Services Research, University of North Carolina at Chapel Hill, Chapel Hill, North Carolina, United States of America; 4 Department of Genome Sciences, University of Washington, Seattle, Washington, United States of America; National Tsing-Hua University: National Tsing Hua University, TAIWAN

## Abstract

Artificial intelligence (AI) has transformative potential in public health, but its impact is limited by models that implicitly prioritize a single stakeholder perspective and do not make explicit and tunable trade-offs between community and clinician endorsement. To address this gap, we introduce Participatory-informed Preference Optimization (PiPrO), a large language model embedding-based calibration framework that generates a single clinical outcome prediction while explicitly accounting for differences between community and physician interpretations of the same scenario. PiPrO takes as input two embeddings derived from a large language model representing a community-facing context and a physician-facing context. It then applies a shared lightweight feedforward predictor to produce per-stakeholder scores which are then mixed using a single global mixing weight (alpha). Alpha controls how strongly the final prediction reflects the community versus physician responses and is learned using a policy-gradient update driven by an abundant but noisy community text and a sparse and biased physician text. PiPrO reliably learned stable alpha values and a consistent reward signal. Alpha shifts systematically toward physician weighting as community feedback becomes noisier and shifts toward community weighting as physician feedback becomes more biased. Our results suggest PiPrO’s potential to produce more transparent, and context-sensitive AI-driven healthcare recommendations. Future research should validate this approach using real-world community inputs to ensure generalizability and practical impact.

## Author summary

Artificial intelligence tools are increasingly adopted in medicine and public health, but they are often trained to reflect only one viewpoint. In practice, community members and physicians can interpret the same clinical situation differently, and those differences can matter for recommendations that affect care. In this study, we developed a method called Participatory-informed Preference Optimization to help a prediction model account for both perspectives while still producing one final prediction. We tested the method in a simulation study using community-facing and physician-facing versions of the same scenario, and we varied how reliable each source of feedback was. We found that the model learned a stable balance between the two perspectives. It shifted toward physician input when community feedback became less reliable, and toward community input when physician feedback became more biased. These results suggest that health-related artificial intelligence can be designed to make trade-offs between stakeholder perspectives more transparent.

## Introduction

Artificial intelligence (AI) is rapidly changing many aspects of medicine and public health [1]. The application of AI to health is accompanied by several well-documented challenges, including hallucination, bias, and a lack of regulatory oversight [2]. These barriers have been primarily evaluated by healthcare providers and technical experts, leaving a gap in the inclusion of patients and community members [3]. Current AI training processes do not systematically incorporate community perspectives, which is an omission of information that could address real-world public health needs effectively [4].

The field of public health has increasingly recognized the importance of patients and community leaders contributing to and directing research and technology development. This is especially true for contributors to the health of communities that have historically been underrepresented in decision-making [5,6]. Community engagement in public health initiatives can improve relevance of health programs to local needs and values, potentially more effectively addressing health disparities [7].

To address these gaps, we introduce Participatory-informed Preference Optimization (PiPrO). PiPrO is a reinforcement learning methodology designed to incorporate community perspectives. PiPrO is designed to integrate community-derived input alongside clinical expertise via continuous feedback, allowing communities and patients to contribute to care recommendations.

There are four main reasons why PiPrO fills these informational gaps. First, by modeling community preferences and stakeholder expertise, PiPrO mitigates algorithmic biases inherent in AI systems trained on unrepresentative datasets. Second, AI systems aligned with local contexts and patient values have higher acceptability and adoption rates, and PiPrO enables individual communities to directly improve models by reflecting their needs and preferences. For example, community-tailored health messages increase adherence and behavior change [8,9], thereby amplifying the real-world health impact of AI interventions. Third, PiPrO uses stakeholder embeddings and a calibration parameter to fine-tune multi-party responses, offering a computationally simple yet flexible alignment framework that can be easily deployed in a variety of settings. Finally, the structured feedback loops inherent in PiPrO offer both local and general transparency about how community inputs influence AI recommendations, as well as iterative inclusion of community preferences over time. This builds trust in AI systems [10], reduces misinformation [11,12], and ensures accountability [13], all of which are critical components of sustained public engagement and effective public health responses [14].

In this paper, we review relevant literature in AI alignment and community-based participatory research (CBPR), introduce the PiPrO method, and conduct a simulation study to evaluate a proof-of-concept in which simulated patient- and provider-preferences guide joint model calibration. This is meant to represent a wide range of scenarios relevant to medicine and public health. In practice, there is a wide range of discordance levels for interventions between patients and providers, and being able to model this range of responses is useful for designing an internally consistent AI model of healthcare.

## Background: AI-driven participatory approaches in public health

While we are primarily focused on presenting our reinforcement learning framework, key methodological choices were made by building on two separate areas of study: AI alignment and community-based participatory research. In this section, we briefly review these two strands of literature and situate our contribution within both. To further situate our contribution, we also provide examples of past AI tools intended for public health applications that explicitly included community feedback and examples where meaningful conflicts may be important to represent within AI systems.

### Techniques to update algorithms based on feedback

There are already numerous existing techniques to achieve feedback on model performance. These methods include Reinforcement Learning with Human Feedback (RLHF), [15] Direct Preference Optimization (DPO), [16] Kahneman-Tversky Optimization (KTO), [17] Reinforcement Learning with Expert Feedback (RLEF), [18] Iterative Preference Optimization (IPO), [19] and Simultaneous Preference Integration for Neural Networks (SPINN) [20]. Kumar et al. (2024) introduced a framework for language models to incorporate community-specific preferences using data from Reddit subforums, conditioning each model on prepended community-specific identifiers to reflect the distinct preferences of that community [21]. We detail these existing techniques in Table 1 below. To the best of our knowledge, none of these current techniques directly model patient-vs-provider preference tradeoffs in classification tasks.

**Table 1:** Overview of techniques to update algorithms based on feedback.

Alignment Technique	Feedback	Update Rule	Required Data
**PiPrO (Participatory-informed Preference Optimization)**	Community members and expert stakeholders	Calibration model that integrates feedback from community members and experts using a self-attention mechanism and calibration parameter	Latent embeddings representing expert and community preferences, preference labels from community members and experts.
**RLHF (Reinforcement Learning with Human Feedback)**	Individual human raters, through written demonstrations to prompts and ranking of model outputs.	Reinforcement learning (e.g., Proximal Policy Optimization) on a reward model trained on human rankings to predict preferred outputs.	Labeler-written and user-submitted prompts, human labeler responses to prompts for supervised fine-tuning, and ranked model outputs to train the reward model.
**DPO (Direct Preference Optimization)**	Human labelers, through relative quality assessment/pairwise rankings of different model outputs for the same prompt.	Optimization under a loss function using pairwise human preferences	Prompt dataset, initial language model output responses, human-labeler-assessed model outputs for each prompt showing a preferred response
**KTO (Kahneman-Tversky Optimization)**	Human labelers, through binary signals indicating a “good” or “bad” model output	Optimization under a loss function modeling human decision biases (prospect theory)	Input prompts given to the model, the model outputs generated for each prompt, binary feedback from human labelers
**RLEF (Reinforcement Learning with Expert Feedback)**	Clinical or domain experts	RL updating with an expert-feedback-trained reward model	Expert feedback signals as training data
**IPO (Iterative Preference Optimization)**	Human raters for initial critic model training dataset	An iterative evaluation is conducted over multiple rounds by a critic model, that was initially trained on a dataset manually annotated with human preferences	Input text prompts used to generate videos, generated video outputs produced by the foundation model, human pairwise rankings or point-wise scores for generated video outputs
**SPINN (Simultaneous Preference Integration for Neural Networks)**	Feedback collected from the system, monitoring network conditions, device capabilities, and other user/service requirements	A scheduler makes decisions to optimize inference, based on an early-exit policy and CNN splitting policy, with a goal to maximize the capacity of the system and to minimize latency	Input data to be processed by the CNN, system state data, user/service requirements, and CNN model parameters.
**ComPO (Community Preference Optimization)**	Community members (e.g., Reddit users)	DPO conditioned on community context data, optimized to maximize the likelihood of generating outputs preferred by the community	Input prompts (e.g., posts from each subreddit,) community context data, preferred and disliked answers/input
**Dynamic treatment regimes and Q-learning (SAPP-Q-Learning; LUQ‑Learning)**	Patient-level outcomes over time (e.g., survival, quality of life, treatment efficacy, severity of side effects, and patient preference)	Q-learning/ backward induction for estimating stage-specific decision rules, using1) a preference-weighted utility that trades off quality of life vs survival; 2) estimate posterior-sample latent preference weights using a latent/Bayesian model	Longitudinal (multi-stage) treatment and history; patient preference survey responses (to estimate latent preference weights); treatment indicators; multi-stage trajectories (trial/EHR/observational); multi-dimensional outcome
**Clinical RL (FQI: fitted-Q-Iteration; DQN: deep Q network)**	Outcome-based reward signals engineered from clinical objectives (e.g., survival, physiologic stability, information gain, complications), sometimes augmented/derived from clinician behavior via inverse RL.	Optimize a policy/value function over retrospective patient trajectories by iteratively updating Q/value estimates and deriving improved policies (off-policy learning from clinician-generated data; evaluation often via counterfactual/offline policy evaluation or simulated rollouts).	Longitudinal ICU/EHR trajectories with state features (demographics, vitals, labs, severity scores), clinician actions (meds/doses/interventions), timing, and outcomes sufficient to define rewards/terminal endpoints; often large ICU datasets for offline training.
**Reinforcement Learning with Near-Optimal Set-Valued Policies (SVP)**	No explicit preference labels required; “preference” is handled implicitly by (1) reward choice and (2) downstream human selection among near-equivalent actions	Value-based RL with a near-greedy inclusion rule, then evaluate SVP via a worst-case (min-over-set) value; implemented with temporal difference learning/Q-learning–style updates (model-free)	Clinical data that provides standard RL trajectories (state, action, reward, next state); does not require patient vs clinician paired preference signals
**Reinforcement Learning from Human Preferences (PbRL/ DRLHP)**	Human raters provide pairwise comparisons (or rankings) of trajectory segments indicating which behavior is preferred	Learn a reward/utility model from preferences (e.g., Bradley–Terry/ sigmoid likelihood), then optimize the policy via standard RL (policy gradient, actor–critic, TRPO); preferences may be queried actively to reduce uncertainty	Trajectory rollouts, human preference labels over trajectory pairs; no explicit reward function required, but assumes preferences reflect a single latent utility
**Inverse RL**	Implicit expert preference revealed through retrospective clinician actions (demonstrations), assumed to reflect optimal or near-optimal decision making	Learn a latent reward function (e.g., weights over physiological stability, oxygenation, duration on ventilator) via Bayesian IRL, then optimize policies using batch RL under the inferred reward	Retrospective clinical trajectories (state, action, next state), feature-engineered outcomes; assumes clinician behavior encodes a coherent reward and requires sufficient coverage of expert decisions
**RL and multi-agent negotiation approaches for shared decision-making (DDPG-based AutoSDM; intuitionistic-fuzzy agent negotiation)**	Explicit but fuzzy doctor and patient preferences, expressed over multiple decision issues (cost, efficacy, side effects), sometimes including hesitation and uncertainty	Multi-agent negotiation in which doctor and patient agents iteratively exchange offers under an alternating-offer protocol; strategies are optimized either via deep reinforcement learning (e.g., Actor–Critic/ DDPG) or via fuzzy or intuitionistic-fuzzy inference rules with time-discounted concessions, aiming to maximize joint or balanced satisfaction	Structured doctor and patient preference profiles (issue weights, fuzzy or intuitionistic-fuzzy parameters), negotiation protocols, and either simulated or elicited preference data
**Contextual bandits** for **sequential decision-making in Just-In-Time Adaptive Interventions (JITAIs)**	Immediate/proximal outcome (reward) observed after each decision (e.g., steps in next hour; binary success/engagement)	Regret-minimizing online updates (e.g., exploration–exploitation via UCB-style (e.g., LinUCB/SupLinUCB) confidence bounds, Thompson sampling/posterior sampling, or ε/epoch-greedy policy updates)	Repeated decision points with (1) context/tailoring variables (sensor + self-report features), (2) action taken (intervention option), and (3) observed proximal outcome/reward (plus timestamps; optionally offline logged data or micro-randomized trial data for initialization/evaluation)

The combination of dynamic treatment regimes and Q-learning that explicitly incorporates patient preferences has been one well-established line of work in statistics and biostatistics. Early work by Butler et al. [22] formalized preference-aware dynamic treatment regimes by balancing competing outcomes through patient-specific utilities, and more recent methods make preferences over multiple outcomes explicit. Survival Augmented Patient Preference Q-Learning (SAPP-Q-Learning) [23] incorporates patient preferences to trade off quality of life and survival under censoring, while Latent Utility Q-Learning (LUQ-Learning) [24] generalizes this approach by modeling preferences as latent utilities over multi-dimensional outcome vectors with flexible elicitation and strong theoretical guarantees. Unlike PiPrO, however, these methods are designed to optimize treatment policies under patient-specific preferences and do not model explicit, potentially conflicting preference labels from multiple stakeholders (e.g., patients and providers). In addition, these reinforcement learning approaches fail to evaluate classification tasks where such preference tradeoffs are central to the practice of medicine**.**

In clinical settings, reviews of reinforcement learning (RL) for decision support emphasize that RL’s key source of flexibility lies in the reward function, and that altering the reward can fundamentally change the policies an algorithm recommends. [25, 25] This flexibility is frequently framed as an opportunity to reflect patient values and support shared decision-making, since different reward formulations can encode different tradeoffs among clinical outcomes (e.g., short-term physiologic stability versus long-term survival). These clinical RL approaches typically treat preferences as implicitly embedded in a scalar reward or utility rather than modeling explicit, potentially conflicting preference signals from multiple stakeholders (such as patients and clinicians) on the same decision task.

Set-valued policy RL (near-optimal SVP/ “clinician-in-the-loop” RL) learns, for each clinical choice, a *set* of actions whose expected cumulative rewards are provably near-equivalent rather than simply a single best action. By relaxing the objective from strict optimality to ζ-optimality, these methods identify multiple treatment options that perform similarly under a scalar reward (e.g., survival), enabling clinicians or patients to incorporate additional considerations—such as side effects, invasiveness, cost, or individual preferences—when selecting among them [26, 27]. The approach is typically implemented using value-based, model-free reinforcement learning with a near-greedy inclusion rule and worst-case evaluation over the action set, providing theoretical guarantees under mild conditions. While this framework supports human-in-the-loop decision making by preserving flexibility at deployment time, it does not explicitly model or learn from stakeholder-specific preference signals, treating preferences as external to the learning process rather than as structured feedback integrated into the algorithm itself. In addition, it does not allow for selection of actions that are suboptimal, only selection among otherwise equivalent choices on the basis of orthogonal considerations.

In the computer science literature, there is a broad tradition of preference-based reinforcement learning (PbRL) that relaxes the assumption of a fully specified reward function, defining learning objectives through preference comparisons over states, actions, or trajectory segments. [28] Classic work shows that complex behaviors can be learned by fitting a latent reward or utility model from (often non-expert) human pairwise comparisons and then optimizing policies against that learned objective, substantially reducing the need for hand-engineered rewards. A general methodological survey formalizes PbRL as solving RL problems using qualitative preference feedback and categorizes approaches by the type of feedback, the learned representation (policy, preference model, or utility), and the associated optimization strategy. More recent work in treatment recommendation similarly applies preference-based RL to learn rewards that encode tradeoffs across multiple clinical goals (e.g., efficacy versus side effects), primarily in simulation. [29] Compared with PiPrO, these approaches typically collapse all feedback into a single latent utility or reward signal and do not explicitly represent, calibrate, or reconcile simultaneous (and not necessarily consistent) preferences, which potentially conflict with preference signals from different stakeholders.

A parallel strand of work treats clinician behavior itself as a revealed preference signal and uses inverse reinforcement learning (IRL) to infer the latent reward functions that appear to guide practice. In ICU settings, for example, Yu et al. infer clinicians’ implicit tradeoffs in ventilation weaning and sedative dosing from retrospective trajectories, and related reviews frame this approach as learning “clinician preference” by interpreting observed decisions as reward-maximizing behavior [29]. This line of work conceptualizes preference as a single, implicit provider objective recovered from data, without jointly incorporating patient-stated preferences on the same decision tasks, or providing an explicit mechanism to balance or calibrate potentially conflicting patient–provider preference signals under noisy feedback.

There is a more direct “patient vs. doctor preferences” literature that models shared decision-making explicitly as a negotiation problem and uses learning-based or rule-based strategies to reach mutually acceptable treatment plans. In this line of work, Chen et al. (2025) [30] propose an RL-based automated negotiation framework in which doctor and patient agents express preferences over multiple treatment dimensions (including side effects) and iteratively improve negotiation strategies via deep reinforcement learning, while related frameworks employ fuzzy or intuitionistic-fuzzy agent negotiation to manage preference uncertainty and concession behavior. [31] Collectively, these approaches treat preference divergence as a core challenge and seek efficient, fair agreements through sequential offer exchange. However, these methods are negotiation-centric, not on calibrating a predictive model’s outputs using simultaneous, heterogeneous preference signals from multiple stakeholders.

Another related literature that frames personalization and preference alignment as a contextual bandit problem, particularly in just-in-time adaptive interventions (JITAIs). In this line of work, Tewari and Murphy (2017) [32] formalize contextual bandits as a framework for sequential decision-making in which interventions are selected based on time-varying contextual information (e.g., stress, location, physiological signals) and updated using proximal outcome feedback, with the goal of minimizing regret while balancing exploration and exploitation. These approaches emphasize online learning of context-dependent decision rules under uncertainty and have been widely studied under stochastic, adversarial, and partially non-stationary settings. Compared to PiPrO, standard contextual bandit formulations typically assume a single implicit reward signal and do not explicitly model or reconcile preference feedback from multiple stakeholder groups.

### Community-based participatory research (CBPR)

CBPR is a model which explicitly aims to make community members equal partners in research.[7,33,34] CBPR projects involve community partners at all stages – from setting the research agenda to designing interventions and disseminating results – and facilitates two-way knowledge exchange between community and researchers [35,36]. Such approaches have been shown to expand the reach of health interventions and influence policies to eliminate disparities.[37–39] Overall, patient and community participation is seen as a catalyst for more effective and equitable public health outcomes, particularly for marginalized groups who often face unique barriers and health inequities [40,41]. PiPrO can serve as one tool to enable faster and easier integration of community feedback into AI development for intervention design so that CBPR-based interventional work is more effective.

### Past examples of AI development with community feedback loops

Including feedback from communities in AI tools is not new; indeed it is broadly essential to many reinforcement learning techniques used to fine-tune models (although this is not typically considered community engagement). Prior health-related AI projects have solicited community input in myriad ways. We now detail examples where community input was a key driver of AI development and application in public health.

The first group of examples focus on using AI tools and community engagement to improve prediction of health outcomes. The improvements in prediction involve improvements in timeliness and accuracy. Participatory disease surveillance systems, such as the “Global Flu View” platform [42], aggregate self-reported symptoms from community members to detect outbreaks.These approaches have promise to detect signs of outbreaks earlier than traditional clinic-based reporting [43]. The FAITH! Trial (Fostering African-American Improvement in Total Health) developed an AI tool to improve cardiovascular health through community-based participatory methods [44] and achieved an area-under-curve (AUC) of 0.89 for detecting reduced ejection fraction [45]which was notable since AI-based screening tools trained in academic settings typically perform poorly in real-world populations due to lack of representative data [46]. In environmental health, the “Smell Pittsburgh” initiative [47]enabled residents used sensors and reported pollution odors to generating a dataset of environmental indicators via AI prediction.[48] In the RISE project, community members and scientists collaboratively annotated images of industrial smoke plumes, which were then used to train an AI model to automatically recognize pollution events [49].

AI chatbots for health have been developed with user-centered design, particularly those focused on involving users in testing [50]. A randomized controlled trial demonstrated that the Wysa chatbot, which employs a user-centered AI approach, significantly reduced symptoms of depression and anxiety in individuals with chronic conditions like arthritis and diabetes.[51] Patient-in-the-loop approaches enable further automated model refinement beyond the developer-centric approach that is currently standard [52]. However, the term “patient-in-the-loop” encompasses a range of methods and implementations - and requires more evidence to establish the generalizability and efficacy of these approaches across different healthcare contexts. It also inherently situates researchers as designers and implementers of study questions, with patients acting as alternative model inputs rather than true collaborators in the answering process.

### Examples where further method development would add value

We propose two illustrative use cases where a method such as PiPrO that aims to represent different preferences and perspectives might be particularly helpful. We anticipate that even more use cases will arise in this fast-moving field.

*Addressing conflicts in preferences:* Patients and health providers often rank the same treatments (e.g., drugs) differently. For example, a discrete choice experiment examining the relative importance of six attributes related to lipid-lowering drugs showed that patients prioritized the mode and frequency of drug administration as the second and third most important factors only after the minimization of liver damage risk, while providers ranked these attributes as the two least significant out of the six. [53] Neither perspective is wrong, but they reflect different priorities.

Standard modeling approaches collapse these differences into an average. When patient and provider preferences are pooled together, the model learns what is most often preferred. However, the model cannot explain disagreement, cannot identify which types of cases drive maximal disagreement, and cannot adjust its recommendations based on whose priorities matter more in a given context.

PiPrO is designed to represent this issue directly. At a high level, it treats drug choice as a problem where multiple preference patterns coexist, rather than assuming a single consensus ranking. It allows the model to recognize that certain types of cases tend to follow one set of priorities (e.g., side-effect sensitivity) while other cases follow another (e.g., efficacy dominance), and to indicate which pattern is most influential for a given recommendation. Importantly, it makes the trade-off between patient- and provider-oriented priorities explicit and adjustable, rather than hiding it inside a single opaque prediction.

*Addressing Algorithmic Bias and Hallucinations:* The integration of AI into digital tools meant to improve health brings ethical considerations such as algorithmic bias, the potential for misinformation, privacy and data security, and questions of accountability and transparency [2].

Algorithmic bias is a well-documented risk in healthcare AI. [54] AI systems trained on data that under-represents certain populations often performs worse for those groups because large language models tend to reflect majority voices present in their training data. [55] Alessandra Bazzano and colleagues (2025) argue that without community engagement, adopting AI in public health exacerbates health inequities, whereas designing AI with community input helps address bias and risks upfront [56].

Involving the community in both development and dissemination of AI can enhance trust and reduce skepticism [57]. This may be particularly true in the detection of plausible but incorrect health information (“hallucinations”) from the AI [58,59]. Such misinformation, if undetected, threatens patient safety [60]. Community feedback loops can serve as an error-correction mechanism [61]. When multiple community members flag a response as inaccurate or harmful, the platform can institute an automatic quarantine of that content until an adjudication process (led by community-elected moderators or domain experts) is completed.

## Methods

### Framework overview

We propose a machine learning framework aimed at improving health care recommendation accuracy by explicitly accounting for the fact that different stakeholders may endorse different recommendations for the same clinical scenario. In the model, each training case has a single target clinical outcome (for example, an adherence outcome), and the input is represented using two separate compact feature sets (“embeddings”) for the same case derived from a large language model. One embedding represents a community-facing view of the scenario, and the other represents a physician-facing view.

PiPrO generates an intermediate prediction from each embedding stream and then combines them using a single, interpretable global mixing weight, which we refer to as **alpha**. Conceptually, alpha functions as a “dial” that determines how strongly the final recommendation reflects the community-based signal versus the physician-based signal. Alpha is learned from feedback rather than being fixed in advance, so the model can adaptively balance these two alignment objectives during training while simultaneously training a single “context to prediction” task by sharing weights across stakeholders, such that the only difference in interpretation on a per-stakeholder basis is the context embedding differences between providers. This enables straightforward interpretation of single stakeholder feedback while building a system that can jointly account for everyone’s needs.

### Model architecture

In this section we will describe the model architecture at a high level.

PiPrO is implemented as a feedforward network with shared weights. This feedforward network is applied separately to the community and physician embeddings to produce two real values scores - one per embedding. Each forward pass is intentionally lightweight: it uses a fully connected layer followed by batch normalization, a rectified linear activation, dropout regularization, and a final fully connected output layer. The hidden layer size is two units, chosen based on the two different stakeholders in the network. Thus, first layer can be thought of as summarization, and the second as co-learning.

The model then forms a single combined score by taking a linear alpha-weighted blend of the two stack outputs, then converts that combined score into a final predicted probability for the target outcome. This architecture scales straightforwardly to more or fewer stakeholders, including stakeholders who occupy the same roles (e.g., two community feedback or two physician feedback providers), including missing predictions on a per-item level.

### Calibration and feedback loop

After producing the predicted probability, PiPrO updates the shared weights of the prediction networks and the global mixing weight alpha using a combined supervised and feedback-driven learning procedure.

The parameter alpha is updated by combining the shared fully connected networks that include both community and physician responses. The two predicted responses are combined into a single reward using the alpha weighting scheme described above. Clinically, this means that alpha shapes both the final recommendation and the contribution of stakeholder endorsement to the learning signal. Alpha is learned with a reinforcement-learning style policy update. The algorithm further samples candidate alpha values per epoch from a Beta probability distribution

We incorporate two stability mechanisms. First, our PiPrO implementation uses a warmup period of 100 epochs in which only the supervised prediction networks are trained; alpha begins updating only after this warmup is complete. Second, it includes a mild regularization term that discourages alpha from collapsing to extreme values unless the endorsement data consistently supports that choice by adding to the loss value for alpha far from equal contribution.

### Data generation

We generated a simulated dataset of 10,000 scenarios in which each case has a single underlying target outcome (for example, overall medication adherence) and two fixed-length numeric representations (“embeddings”) of the same scenario derived from textual representations generated from the llama3.1 model: one intended to reflect a community-facing view and one intended to reflect a physician-facing view. These paired embeddings are the only inputs provided to the model.

During training, we simulate two endorsement streams to reflect how feedback differs by stakeholder group. Community feedback is available for every case but is imperfect: it matches the underlying outcome most of the time, with a specified probability of being randomly inverted to represent noisy or inconsistent community endorsement. Physician feedback is higher value but limited: at each training step, only a randomly selected subset of cases is “queried” for expert input, and the physician label is intentionally biased toward high adherence (meaning it is sometimes set to “adherence” regardless of the underlying outcome). The model’s prediction is converted into a yes/no recommendation using a fifty percent cutoff and scored as agreement versus disagreement with each stakeholder. In experiments, we vary the community noise level, the number of physician queries per batch, and the strength of physician bias to stress-test the calibration mechanism under realistic feedback constraints.

### Evaluation metrics and visualization

Following model training, we assessed performance on the held-out test set. Outcome prediction performance was summarized with mean squared error (average squared difference between the predicted outcome probability and the target outcome) and mean absolute error (average absolute difference). We report these metrics for PiPrO (with learned alpha). To reflect run-to-run variability, we repeated experiments across multiple random seeds and visualized uncertainty over epochs using aggregated curves (with confidence bands).

To characterize training dynamics and the calibration process, we generated a set of standard diagnostic plots. First, we plotted epoch-by-epoch learning curves for mean squared error and mean absolute error on the held-out set, alongside the baseline reference. Second, we plotted the trajectory of the learned global mixing weight, summarizing alpha across seeds using the median and interquartile range; when available, we also included a reference “optimal alpha” computed from an offline alpha sweep for the same experimental condition. Third, to visualize how model predictions track each stakeholder’s interpretation, we plotted stakeholder-specific mean absolute error curves for the community signal and for the physician signal over training (noting that physician feedback is sparse by design and only present for a queried subset during training). Fourth, we plotted the policy-gradient reward signal used to update alpha, to document the magnitude and stability of the feedback signal driving calibration. Finally, we performed two post-hoc visualizations to contextualize alpha: (i) an alpha sweep plot that evaluates outcome prediction error across a grid of fixed alpha values under multiple noise/bias conditions in comparison to the true data, and (ii) a bias–noise landscape heatmap that summarizes the learned alpha values across combinations of community noise and physician bias settings.

### Ethics statement

This study is a simulation study of an algorithmic framework. Thus it does not involve live participants and does not require approval by an Institutional Review Board for a statistical simulation involving no data collection.

### Training conditions

Training was performed using NVIDIA L40S GPUs. Each synthetic clinical scenario was converted into an embedding using the Llama 3.1 language model run locally via Ollama in Python.

## Results

**Fig 1:**
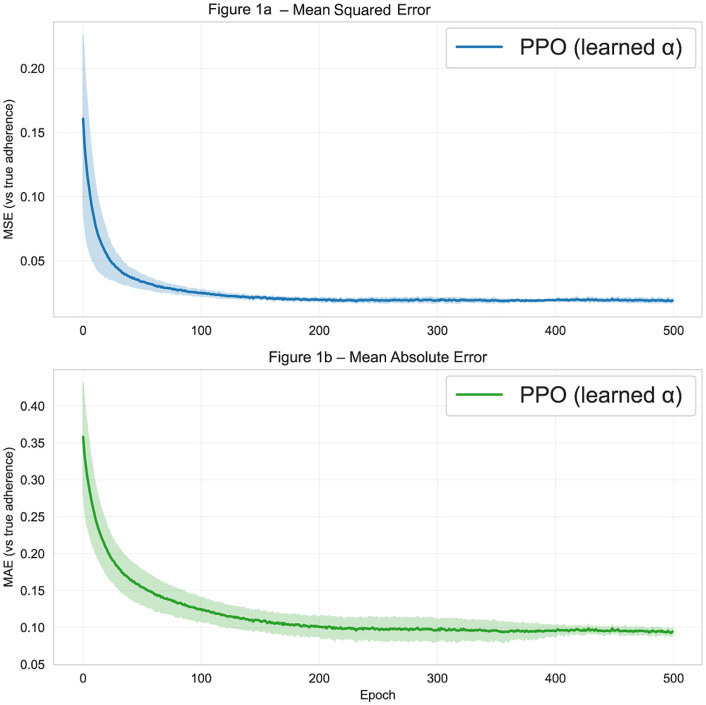
Model learns to predict true adherence.

Fig 1 shows that across epochs, the PiPrO model with policy-gradient alpha learning showed a rapid initial reduction in outcome prediction error followed by a clear plateau. Mean squared error dropped sharply from the initial value (approximately 0.16) to a stable range near 0.02 by roughly 150–200 epochs, with uncertainty bands narrowing over time, indicating reduced variability across seeds as training progressed. Mean absolute error similarly declined from approximately 0.37 to a stable range near 0.09–0.10 by the end of training.

**Fig 2:**
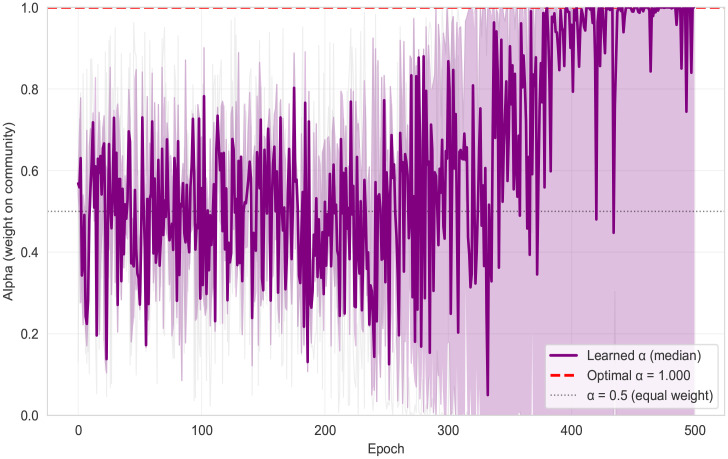
Learning ɑ (σ_comm = 0.0, β_phys = 0.0).

Fig 2 shows that the learned alpha exhibited substantial exploration early in training in an example of 3 parameter combinations, fluctuating around the equal-weight reference (0.5) with a wide interquartile range across seeds. After the period where alpha began to change systematically, the median alpha increased steadily and then saturated near the upper boundary (approaching 1.0) late in training, indicating convergence toward a community-dominant mixing regime in this parameter slice. The run-level variability was highest during the transition period and narrowed once alpha approached its terminal regime, consistent with stabilization of the policy update.

**Fig 3:**
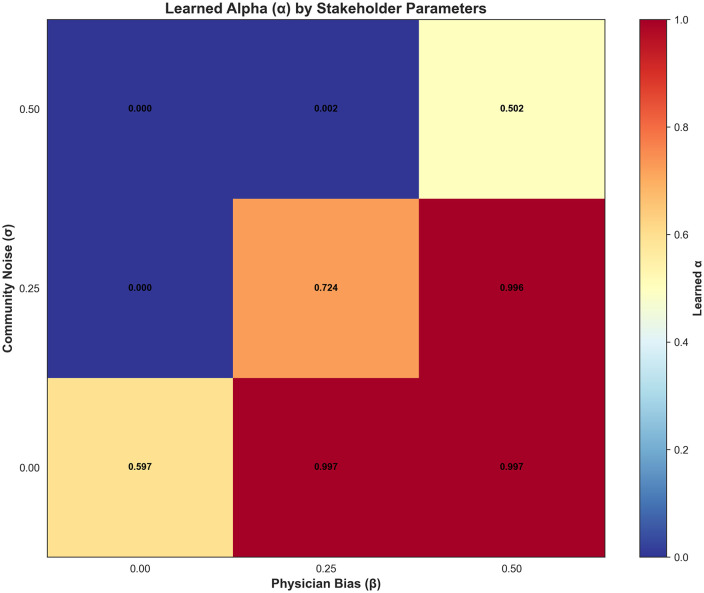
Alignment with community vs. physician interpretations.

Fig 3 presents a heatmap summarizing the final learned value of alpha across a grid of stakeholder-feedback conditions. The horizontal axis varies physician bias (the degree to which simulated physician feedback is pushed toward high adherence), and the vertical axis varies community noise (the probability that community feedback is randomly flipped). Each cell reports the learned alpha value for that parameter combination, and the color scale maps alpha from 0 (physician-dominant mixing) to 1 (community-dominant mixing). The main scientific takeaway from Fig 3 is that the learned mixing weight alpha adapts in a directionally appropriate way to the relative reliability of the two feedback streams: when the community signal becomes noisier, the policy shifts alpha downward (placing more weight on the physician stream), and when the physician signal becomes more biased, the policy shifts alpha upward (placing more weight on the community stream). When both stakeholder signals are degraded at the same time, the learned alpha returns toward an approximately equal-weight compromise rather than collapsing to either extreme.

**Fig 4:**
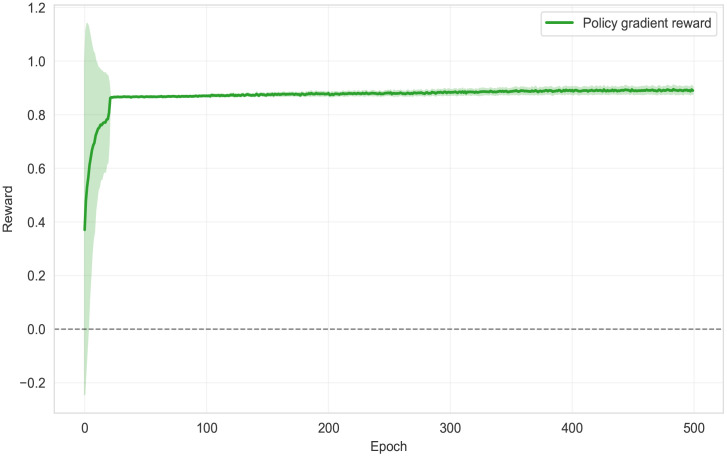
Reward signal guiding ɑ learning.

Fig 4 shows that the policy-gradient reward signal increased sharply during the earliest epochs (from approximately 0.37 to the high 0.8s within the first few dozen epochs) and then remained stably positive for the remainder of training, with a slow upward drift toward approximately 0.89–0.90. The confidence band was wide early, reflecting high across-seed variability when the model and policy are immature, and then narrowed substantially as training progressed, indicating a stable reward landscape once the system reached its steady-state regime.

**Fig 5:**
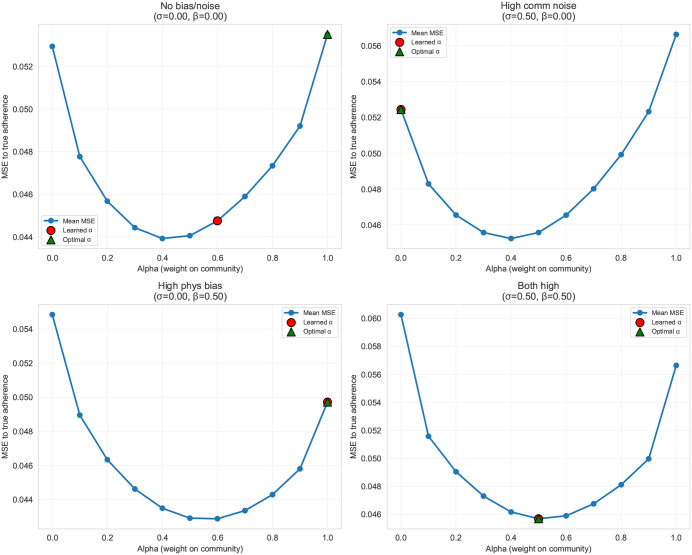
Alpha sweep: finding optimal bias-variance balance.

Fig 5 shows that across all four sweep conditions, outcome prediction error as a function of fixed alpha was consistently convex (U-shaped), with the lowest mean squared error occurring at intermediate alpha values (roughly in the 0.4–0.6 range) rather than at the extremes. In contrast, the alpha selected by the learned policy tended to move toward boundary solutions (near 0 or near 1) when only one feedback stream was degraded (high community noise or high physician bias), while it remained near the center (approximately 0.5) when both streams were simultaneously degraded. Operationally, these sweeps demonstrate that the alpha values favored by the feedback-driven policy can diverge from the alpha values that minimize squared error to the true outcome, highlighting a measurable trade-off between optimizing the endorsement-driven objective and optimizing pure outcome prediction performance.

### Ethical considerations in for the Implementation of PiPrO

Calibrating preferences based heavily on community feedback might inadvertently reinforce existing biases, echo chambers, or misinformation present within specific communities, particularly without validation mechanisms or using training strategies not incorporating measures of trust. In this section, we discuss how involving communities through approaches like PiPrO can mitigate or exacerbate risks such as bias and misinformation, and what safeguards are needed to help ensure responsible AI use. PiPrO can also enable inclusion of expert opinions into mostly patient-derived modeling efforts as a means of reducing the effect of misinformation as well.

Kumar et al. (2024) emphasize that while personalizing AI to community preferences has benefits, it must be done with safeguards to avoid creating echo chambers that entrench the community’s own biases.[21] In practice, this might translate to requiring and reweighting diverse community representation in input datasets, forming community advisory boards for AI projects, and continuously monitoring algorithm outcomes. As described by Kumar et al (2024), one approach is tiered training on two major sources of knowledge. Tier 1 consists of universal best practices (e.g., peer-reviewed studies, WHO guidelines) to anchor the model in evidence-based standards. Tier 2 incorporates local data (e.g., health department protocols, regional dialect or cultural knowledge). The system could also use Retrieval-Augmented Generation to ground the AI responses in information from community trusted sources [62]. A future PiPrO implementation could integrate an evidence check; the present simulation addresses only synthetic patient and provider label information and does not incorporate external knowledge bases or topic-specific expertise.

AI-based platforms are susceptible to adversarial misuse [63]. This can arise through manipulative prompts designed to elicit harmful content or through data poisoning attacks, in which false user feedback accumulates to misalign the model [64]. Community participation can indeed mitigate adversarial threats through collective oversight, but it can also inadvertently introduce risks if not carefully managed [65]. Without robust safeguards, open community input can be exploited for data poisoning or coordinated misinformation [66].

## Conclusion

In this work, we introduced a calibrated four‑head ensemble that aims to balance patient and provider preferences in a synthetic decision task. Our proof-of-concept demonstrated substantial improvements in aligning recommendations with diverse stakeholder perspectives, particularly when preferences diverge. The simulated community and provider feedback showed the potential for effective refinement of model behavior. This approach holds promise for improving the effectiveness and acceptability of AI-enabled digital health tools.

This work has limitations. The current approach relies on synthetic data generation. Real-world data might contain complex nuances and interactions that synthetic embeddings do not capture, and will likely require larger training (or pre-training) sample sizes to obtain robust results. The simplified binary representation (patient/provider preferences) restricts capturing more nuanced or gradient preferences frequently seen in clinical and community contexts. Feedback loops in this study are simulated, assuming probabilistic interactions. Actual feedback from patients and providers may vary considerably in quality, frequency, and type, even over time. The next step is to generate a community-relevant, stakeholder-aware dataset that allows testing of multiple stakeholder real world problems for AI. To explore human-centered validation in the drug-versus-lifestyle treatment example, we would deploy our framework in a real clinical workflow where clinicians and patients review the recommendation, record their preferences and rationale, and then track downstream patient-centered outcomes (e.g., adherence, symptom control, adverse events, and patient-reported burden) to test whether PiPrO improves welfare-relevant endpoints when preferences conflict. A prospective extension of this application would involve the randomization (or the use of a stepped-wedge rollout) of PiPrO recommendations to establish causal effects and assess usability and trust among patients and clinicians.

The ensemble method introduces computational complexity and may be challenging to scale to larger, high-dimensional datasets or real-time inference situations. We explored only a single hyperparameter (α). Other potentially influential hyperparameters (e.g., network architecture details, learning rates, attention mechanism parameters) were not systematically explored, potentially limiting performance optimization.

Partnering with communities and trusted local leaders is a recurring recommendation in addressing health misinformation in public health [57]. In practice, this means that the development of AI interventions should not occur in isolation. Continuous dialogue with the community must guide what the AI does and how it does it, pointing toward a future of “community-engaged AI development” in public health. Our calibration mechanism, once combined with genuine community feedback and co-design, could illustrate one pathway toward this vision of “community-engaged AI development”, demonstrating how iterative community input can dynamically refine model recommendations, actively balancing community preferences and clinical guidelines.

## Supporting information

S1 File Reward function. (PDF)

S2 File Algorithm. (PDF)

S3 File Model architecture diagram. (PDF)

S4 File Embedding correlations. (PDF)

## References

[1] Dangi RR, Sharma A, Vageriya V. Transforming healthcare in low-resource settings with artificial intelligence: Recent developments and outcomes. Public Health Nurs. 2025;42(2):1017–30. doi: 10.1111/phn.13500 39629887

[2] Templin T, Perez MW, Sylvia S, Leek J, Sinnott-Armstrong N. Addressing 6 challenges in generative AI for digital health: A scoping review. PLOS Digit Health. 2024;3(5):e0000503. doi: 10.1371/journal.pdig.0000503 38781686 PMC11115971

[3] Nadarzynski T, Knights N, Husbands D, Graham CA, Llewellyn CD, Buchanan T, et al. Achieving health equity through conversational AI: A roadmap for design and implementation of inclusive chatbots in healthcare. PLOS Digit Health. 2024;3(5):e0000492. doi: 10.1371/journal.pdig.0000492 38696359 PMC11065243

[4] Barony Sanchez RH, Bergeron-Drolet L-A, Sasseville M, Gagnon M-P. Engaging patients and citizens in digital health technology development through the virtual space. Front Med Technol. 2022;4:958571. doi: 10.3389/fmedt.2022.958571 36506474 PMC9732568

[5] Sofolahan-Oladeinde Y, Mullins CD, Baquet CR. Using community-based participatory research in patient-centered outcomes research to address health disparities in under-represented communities. J Comp Eff Res. 2015;4(5). 10.2217/cer.15.31 26436953

[6] Cyril S, Smith BJ, Possamai-Inesedy A, Renzaho AMN. Exploring the role of community engagement in improving the health of disadvantaged populations: A systematic review. Glob Health Action. 2015;8:29842. doi: 10.3402/gha.v8.29842 26689460 PMC4685976

[7] Morales-Garzón S, Parker LA, Hernández-Aguado I, González-Moro Tolosana M, Pastor-Valero M, Chilet-Rosell E. Addressing health disparities through community participation: A scoping review of co-creation in public health. Healthcare (Basel). 2023;11(7):1034. doi: 10.3390/healthcare11071034 37046961 PMC10094395

[8] Kukula VA, Odopey S, Arthur E, Odonkor G, Awini E, Adjei A, et al. Understanding health worker and community antibiotic prescription-adherence practices for acute febrile illness: A nested qualitative study in the Shai-Osudoku District of Ghana and the development of a training-and-communication intervention. Clin Infect Dis. 2023;77(Suppl 2):S182-90. 10.1093/cid/ciad327 PMC10368414 37490740

[9] Vedanthan R, Kamano JH, DeLong AK, Naanyu V, Binanay CA, Bloomfield GS, et al. Community health workers improve linkage to hypertension care in western Kenya. J Am Coll Cardiol. 2019;74(15):1897–906. 31487546 10.1016/j.jacc.2019.08.003 PMC6788970

[10] Stroud AM, Minteer SA, Zhu X, Ridgeway JL, Miller JE, Barry BA. Patient information needs for transparent and trustworthy cardiovascular artificial intelligence: A qualitative study. PLOS Digit Health. 2025;4(4):e0000826. doi: 10.1371/journal.pdig.0000826 40258073 PMC12011294

[11] Kisa S, Kisa A. A Comprehensive Analysis of COVID-19 Misinformation, Public Health Impacts, and Communication Strategies: Scoping Review. J Med Internet Res. 2024;26:e56931. doi: 10.2196/56931 39167790 PMC11375383

[12] Jin SL, Kolis J, Parker J, Proctor DA, Prybylski D, Wardle C, et al. Social histories of public health misinformation and infodemics: Case studies of four pandemics. Lancet Infect Dis. 2024;24(10):e638–46. doi: 10.1016/S1473-3099(24)00105-1 38648811

[13] Rice BT, Rasmus S, Onders R, Thomas T, Day G, Wood J, et al. Community-engaged artificial intelligence: An upstream, participatory design, development, testing, validation, use and monitoring framework for artificial intelligence and machine learning models in the Alaska Tribal Health System. Front Artif Intell. 2025;8:1568886. doi: 10.3389/frai.2025.1568886 40260415 PMC12009764

[14] Sadeghi Z, Alizadehsani R, Cifci MA, Kausar S, Rehman R, Mahanta P, et al. A review of explainable artificial intelligence in healthcare. Comput Electr Eng. 2024;118(109370):109370.

[15] Ouyang L, Wu J, Jiang X, Almeida D, Wainwright CL, Mishkin P. Training language models to follow instructions with human feedback. Neural Inf Process Syst. 2022;:27730–44. doi: abs/2203.02155

[16] Rafailov R, Sharma A, Mitchell E, Ermon S, Manning CD, Finn C. Direct preference optimization: your language model is secretly a reward model. 2023. http://arxiv.org/abs/2305.18290

[17] Ethayarajh K, Xu W, Muennighoff N, Jurafsky D, Kiela D. KTO: Model alignment as prospect theoretic optimization. 2024. http://arxiv.org/abs/2402.01306

[18] Liu S, See KC, Ngiam KY, Celi LA, Sun X, Feng M. Reinforcement learning for clinical decision support in critical care: Comprehensive review. J Med Internet Res. 2020;22(7):e18477. doi: 10.2196/18477 32706670 PMC7400046

[19] Yang X, Tan Z, Nie X, Li H. IPO: Iterative Preference Optimization for Text-to-Video Generation. 2025. http://arxiv.org/abs/2502.02088

[20] Laskaridis S, Venieris SI, Almeida M, Leontiadis I, Lane ND. SPINN: Synergistic progressive inference of neural networks over device and cloud. Proceedings of the 26th Annual International Conference on Mobile Computing and Networking, 2020. https://dl.acm.org/doi/10.1145/3372224.3419194

[21] Community Preferences for Language Model Personalization. https://arxiv.org/html/2410.16027v1. Accessed 2025 February 18.

[22] Butler EL, Laber EB, Davis SM, Kosorok MR. Incorporating Patient Preferences into Estimation of Optimal Individualized Treatment Rules. Biometrics. 2018;74(1):18–26. doi: 10.1111/biom.12743 28742260 PMC5785589

[23] Zhong Y, Wang C, Wang L. Survival augmented patient preference incorporated reinforcement learning to evaluate tailoring variables for personalized healthcare. Stats. 2021;4(4):776–92.

[24] Zitovsky JP, Zou Y, Wilson L, Kosorok MR. A flexible framework for incorporating patient preferences into Q-learning. 2023. 10.48550/ARXIV.2307.12022

[25] Liu S, See KC, Ngiam KY, Celi LA, Sun X, Feng M. Reinforcement Learning for Clinical Decision Support in Critical Care: Comprehensive Review. J Med Internet Res. 2020;22(7):e18477. doi: 10.2196/18477 32706670 PMC7400046

[26] Tang S, Modi A, Sjoding MW, Wiens J. Clinician-in-the-loop decision making: reinforcement learning with near-optimal set-valued policies. 2020. http://arxiv.org/abs/2007.12678

[27] Christiano P, Leike J, Brown TB, Martic M, Legg S, Amodei D. Deep reinforcement learning from human preferences. 2017. 10.48550/ARXIV.1706.03741

[28] Wirth C, Akrour R, Neumann G, Fürnkranz J. A survey of preference-based reinforcement learning methods. J Mach Learn Res. 2017;18(136):136:1-136:46.

[29] Yu C, Liu J, Zhao H. Inverse reinforcement learning for intelligent mechanical ventilation and sedative dosing in intensive care units. BMC Med Inform Decis Mak. 2019;19(Suppl 2):57. doi: 10.1186/s12911-019-0763-6 30961594 PMC6454602

[30] Chen X, Lu P, Liu Y, Hong FP. Deep deterministic policy gradient-based automatic negotiation framework for shared decision-making. Sci Rep. 2025;15(1):26337. 40685430 10.1038/s41598-025-11001-1 PMC12277425

[31] Lu P, Lu H, Wei Y, Dai B, Lin K, Wen S. An intuitionistic fuzzy automated negotiation model for personalized and efficient shared decision-making. Sci Rep. 2025;15(1):44000. doi: 10.1038/s41598-025-27633-2 41408119 PMC12711877

[32] Tewari A, Murphy SA. From ads to interventions: Contextual bandits in mobile health. In: Mobile Health. Cham: Springer International Publishing; 2017. 495–517.

[33] Carlisle S. Tackling health inequalities and social exclusion through partnership and community engagement? A reality check for policy and practice aspirations from a Social Inclusion Partnership in Scotland. Critical Public Health. 2010;20(1):117–27. doi: 10.1080/09581590802277341

[34] Wallerstein N, Duran B. Community-based participatory research contributions to intervention research: the intersection of science and practice to improve health equity. Am J Public Health. 2010;100 Suppl 1(Suppl 1):S40-6. doi: 10.2105/AJPH.2009.184036 20147663 PMC2837458

[35] South J, Phillips G. Evaluating community engagement as part of the public health system. J Epidemiol Community Health. 2014;68(7):692–6. doi: 10.1136/jech-2013-203742 24671849

[36] Guideline N. Community engagement: improving health and wellbeing and reducing health inequalities. https://www.nice.org.uk/guidance/ng44/resources/community-engagement-improving-health-and-wellbeing-and-reducing-health-inequalities-1837452829381

[37] Wallerstein NB, Duran B. Using community-based participatory research to address health disparities. Health Promot Pract. 2006;7(3):312–23. doi: 10.1177/1524839906289376 16760238

[38] Heimburg D von, Cluley V. Advancing complexity-informed health promotion: A scoping review to link health promotion and co-creation. Health Promot Int. 2021;36(2):581–600. doi: 10.1093/heapro/daaa063 32810227

[39] Leask CF, Sandlund M, Skelton DA, Altenburg TM, Cardon G, Chinapaw MJM, et al. Framework, principles and recommendations for utilising participatory methodologies in the co-creation and evaluation of public health interventions. Res Involv Engagem. 2019;5:2. doi: 10.1186/s40900-018-0136-9 30652027 PMC6327557

[40] Kobetz E, Menard J, Diem J, Barton B, Blanco J, Pierre L, et al. Community-based participatory research in Little Haiti: challenges and lessons learned. Prog Community Health Partnersh. 2009;3(2):133–7. doi: 10.1353/cpr.0.0072 20208260

[41] Mance GA, Mendelson T, Byrd B, Jones J, Tandon D. Utilizing community-based participatory research to adapt a mental health intervention for African American emerging adults. Prog Community Health Partnersh. 2010;4(2):131–40. doi: 10.1353/cpr.0.0112 20543488

[42] Leal Neto O, Paolotti D, Dalton C, Carlson S, Susumpow P, Parker M, et al. Enabling Multicentric Participatory Disease Surveillance for Global Health Enhancement: Viewpoint on Global Flu View. JMIR Public Health Surveill. 2023;9:e46644. doi: 10.2196/46644 37490846 PMC10504624

[43] Artificial Intelligence for Public Health Surveillance in Africa: Applications and Opportunities. https://arxiv.org/html/2408.02575v1. Accessed 2025 April 15.

[44] Harmon DM, Adedinsewo D, Van’t Hof JR, Johnson M, Hayes SN, Lopez-Jimenez F, et al. Community-based participatory research application of an artificial intelligence-enhanced electrocardiogram for cardiovascular disease screening: A FAITH! Trial ancillary study. Am J Prev Cardiol. 2022;12:100431. doi: 10.1016/j.ajpc.2022.100431 36419480 PMC9677088

[45] Brewer LC, Jenkins S, Hayes SN, Kumbamu A, Jones C, Burke LE, et al. Community-based, cluster-randomized pilot trial of a cardiovascular mobile health intervention: Preliminary findings of the FAITH! Trial. Circulation. 2022;146(3):175–90. 35861762 10.1161/CIRCULATIONAHA.122.059046 PMC9287100

[46] Wong A, Otles E, Donnelly JP, Krumm A, McCullough J, DeTroyer-Cooley O, et al. External validation of a widely implemented proprietary sepsis prediction model in hospitalized patients. JAMA Intern Med. 2021;181(8):1065–70. doi: 10.1001/jamainternmed.2021.2626 34152373 PMC8218233

[47] Hsu Y-C, Huang T-H “Kenneth,” Verma H, Mauri A, Nourbakhsh I, Bozzon A. Empowering local communities using artificial intelligence. Patterns (N Y). 2022;3(3):100449. doi: 10.1016/j.patter.2022.100449 35510187 PMC9058901

[48] Hsu YC, Cross J, Dille P, Tasota M, Dias B, Sargent R. Smell Pittsburgh: Engaging community citizen science for air quality. 2019. http://arxiv.org/abs/1912.11936

[49] Hsu YC, Huang TH (K en neth), Hu TY, Dille P, Prendi S, Hoffman R. Project RISE: Recognizing industrial smoke emissions. Proceedings of the Conference on Artificial Intelligence, 2021. 14813–21.

[50] Ma Y, Achiche S, Pomey M-P, Paquette J, Adjtoutah N, Vicente S, et al. Adapting and Evaluating an AI-Based Chatbot Through Patient and Stakeholder Engagement to Provide Information for Different Health Conditions: Master Protocol for an Adaptive Platform Trial (the MARVIN Chatbots Study). JMIR Res Protoc. 2024;13:e54668. doi: 10.2196/54668 38349734 PMC10900097

[51] MacNeill AL, Doucet S, Luke A. Effectiveness of a Mental Health Chatbot for People With Chronic Diseases: Randomized Controlled Trial. JMIR Form Res. 2024;8:e50025. doi: 10.2196/50025 38814681 PMC11176869

[52] Gupta A, Lash MT, Nachimuthu SK. Optimal sepsis patient treatment using human-in-the-loop artificial intelligence. http://arxiv.org/abs/2009.07963. 2020. Accessed 2025 April 15.

[53] Zhang L, Chen J, Cao Z, Zhang M, Ma R, Zhang P, et al. Patient versus physician preferences for lipid-lowering drug therapy: A discrete choice experiment. Health Expect. 2024;27(2):e14043. doi: 10.1111/hex.14043 38590082 PMC11002318

[54] Obermeyer Z, Powers B, Vogeli C, Mullainathan S. Dissecting racial bias in an algorithm used to manage the health of populations. Science. 2019;366(6464):447–53. 31649194 10.1126/science.aax2342

[55] Wong J, Kim J. ChatGPT is more likely to be perceived as male than female. http://arxiv.org/abs/2305.12564. 2023. Accessed 2025 February 18.

[56] Bazzano AN, Mantsios A, Mattei N, Kosorok MR, Culotta A. AI can be a powerful social innovation for public health if community engagement is at the core. J Med Internet Res. 2025;27:e68198. doi: 10.2196/68198 39841529 PMC11799803

[57] Office of the Surgeon General (OSG). WE CAN TAKE ACTION. In: Confronting Health Misinformation: The US Surgeon General’s Advisory on Building a Healthy Information Environment. US Department of Health and Human Services; 2021. 34283416

[58] Ahmad M, Yaramic I, Roy TD. Creating trustworthy LLMs: Dealing with hallucinations in healthcare AI. 2023. https://www.preprints.org/manuscript/202310.1662/v1

[59] Lee P, Bubeck S, Petro J. Benefits, limits, and risks of GPT-4 as an AI chatbot for medicine. N Engl J Med. 2023;388(13):1233–9. 36988602 10.1056/NEJMsr2214184

[60] Asgari E, Montaña-Brown N, Dubois M, Khalil S, Balloch J, Yeung JA, et al. A framework to assess clinical safety and hallucination rates of LLMs for medical text summarisation. NPJ Digit Med. 2025;8(1):274. doi: 10.1038/s41746-025-01670-7 40360677 PMC12075489

[61] Data Quality Awareness: A Journey from Traditional Data Management to Data Science Systems. https://arxiv.org/html/2411.03007v1?utm_source=chatgpt.com. Accessed 2025 April 15.

[62] Yang R, Ning Y, Keppo E, Liu M, Hong C, Bitterman DS. Retrieval-augmented generation for generative artificial intelligence in medicine. 2024. http://arxiv.org/abs/2406.12449 10.1038/s44401-024-00004-1 PMC13354210 42527447

[63] Emerging Cyber Attack Risks of Medical AI Agents. https://arxiv.org/html/2504.03759v1?utm_source=chatgpt.com. Accessed 2025 April 15.

[64] Newaz AI, Haque NI, Sikder AK, Rahman MA, Uluagac AS. Adversarial attacks to machine learning-based smart healthcare systems. 2020. http://arxiv.org/abs/2010.03671

[65] Enhancing guardrails for safe and secure healthcare AI. https://arxiv.org/html/2409.17190v1?utm_source=chatgpt.com. Accessed 2025 April 15.

[66] Mehrabi N, Morstatter F, Saxena N, Lerman K, Galstyan A. A survey on bias and fairness in machine learning. 2019. http://arxiv.org/abs/1908.09635

