# Supplementary material for: Participatory-informed preference optimization (PiPrO): A reinforcement learning simulation study
Source: PLOS Digit Health. 2026 Mar 19;5(3):e0001294. doi: 10.1371/journal.pdig.0001294 (PMC13001916; doi:10.1371/journal.pdig.0001294)
Supplement: S1 File — (PDF) [file pdig.0001294.s001.pdf]

S1 Text. Reward Function

Let $y_i^\star \in [0,1]$ denote the target adherence probability for instance i. The model uses a **single shared** fully-connected stack f_θ:

$$\ell_i^{(\mathrm{comm})} = f_\theta(X_{\mathrm{comm},i}), \qquad \ell_i^{(\mathrm{phys})} = f_\theta(X_{\mathrm{phys},i}).$$

A global mixing weight $\alpha \sim$ Beta (sampled once per minibatch) combines the logits:

$$\ell_i^{(\mathrm{mix})} = \alpha \ell_i^{(\mathrm{comm})} + (1-\alpha)\ell_i^{(\mathrm{phys})} \implies \hat{y}_i = \sigma(\ell_i^{(\mathrm{mix})}). \tag{1}$$

Feedback & Alignment Rewards

- **Community Feedback:** $y_i^{(\mathrm{comm})}$ is the target $y_i^\star$ flipped with probability σ.
- **Physician Feedback:** For a subset I_e of size B_e, $y_j^{(\mathrm{phys})}$ is forced to 1.0 with probability b, else $y_j^\star$. Physician feedback is re-sampled independently for every minibatch.
- **Rewards:** $r_i^{(\mathrm{src})} = 1$ if $\mathrm{sgn}(\hat{y}_i - 0.5) = \mathrm{sgn}(y_i^{(\mathrm{src})} - 0.5)$, else -1.

The total per-instance reward is: $r_i = \alpha r_i^{(\mathrm{comm})} + (1-\alpha) r_i^{(\mathrm{phys})}$.
