# Supplementary material for: Participatory-informed preference optimization (PiPrO): A reinforcement learning simulation study
Source: PLOS Digit Health. 2026 Mar 19;5(3):e0001294. doi: 10.1371/journal.pdig.0001294 (PMC13001916; doi:10.1371/journal.pdig.0001294)
Supplement: S2 File — (PDF) [file pdig.0001294.s002.pdf]

S2 Text. Algorithm

Algorithm 1 PiPrO Training

Require: Embeddings $X_{\text{comm}}, X_{\text{phys}}$, targets $y^{\star}$, σ, B_e, b
Require: `BETA_CONC` $= 4.0$, `PG_LR` $= 0.005$, `MIX_LR` $= 0.01$, EMA $\beta = 0.9$

Initialize shared f_θ, policy logit ϕ, baseline $B \leftarrow 0$
for *epoch* $= 1$ to 200 **do**
  for each minibatch $\mathcal{B}$ **do**
    Compute $\ell^{\text{comm}}, \ell^{\text{phys}} \leftarrow f_\theta(\cdot)$
    $\alpha_0 \leftarrow \sigma(\phi)$
    Sample $\alpha \sim \text{Beta}(\alpha_0 c, (1-\alpha_0)c)$
    Compute $\hat{y}$ using detached α
    $\mathcal{L}_{\text{MSE}} \leftarrow \text{MSE}(\hat{y}, y^{\star}_{\mathcal{B}})$
    Generate $r_i^{(\text{comm})}$ for $i \in \mathcal{B}$
    Sample fresh physician indices in $\mathcal{B}$, generate $r_i^{(\text{phys})}$ (0 elsewhere)
    $r_i \leftarrow \alpha\, r_i^{(\text{comm})} + (1-\alpha)\, r_i^{(\text{phys})}$
    $\bar{r} \leftarrow \text{mean}(r_i)$
    $B \leftarrow \beta B + (1-\beta)\bar{r}$
    $\mathcal{L}_{\text{PG}} \leftarrow -\text{mean}(\log p(\alpha) \cdot r_i)$
    $\mathcal{L}_{\text{mix}} \leftarrow (\alpha - 0.5)^2$
    if *epoch* > 100 **then**
      Update θ, ϕ minimizing $\mathcal{L}_{\text{MSE}} + 0.005\,\mathcal{L}_{\text{PG}} + 0.01\,\mathcal{L}_{\text{mix}}$
    else
      Update θ using $\mathcal{L}_{\text{MSE}}$
