# Supplementary figures and images for: Participatory-informed preference optimization (PiPrO): A reinforcement learning simulation study
Source: PLOS Digit Health. 2026 Mar 19;5(3):e0001294. doi: 10.1371/journal.pdig.0001294 (PMC13001916; doi:10.1371/journal.pdig.0001294)

**S3 Figure.** Model Architecture Diagram

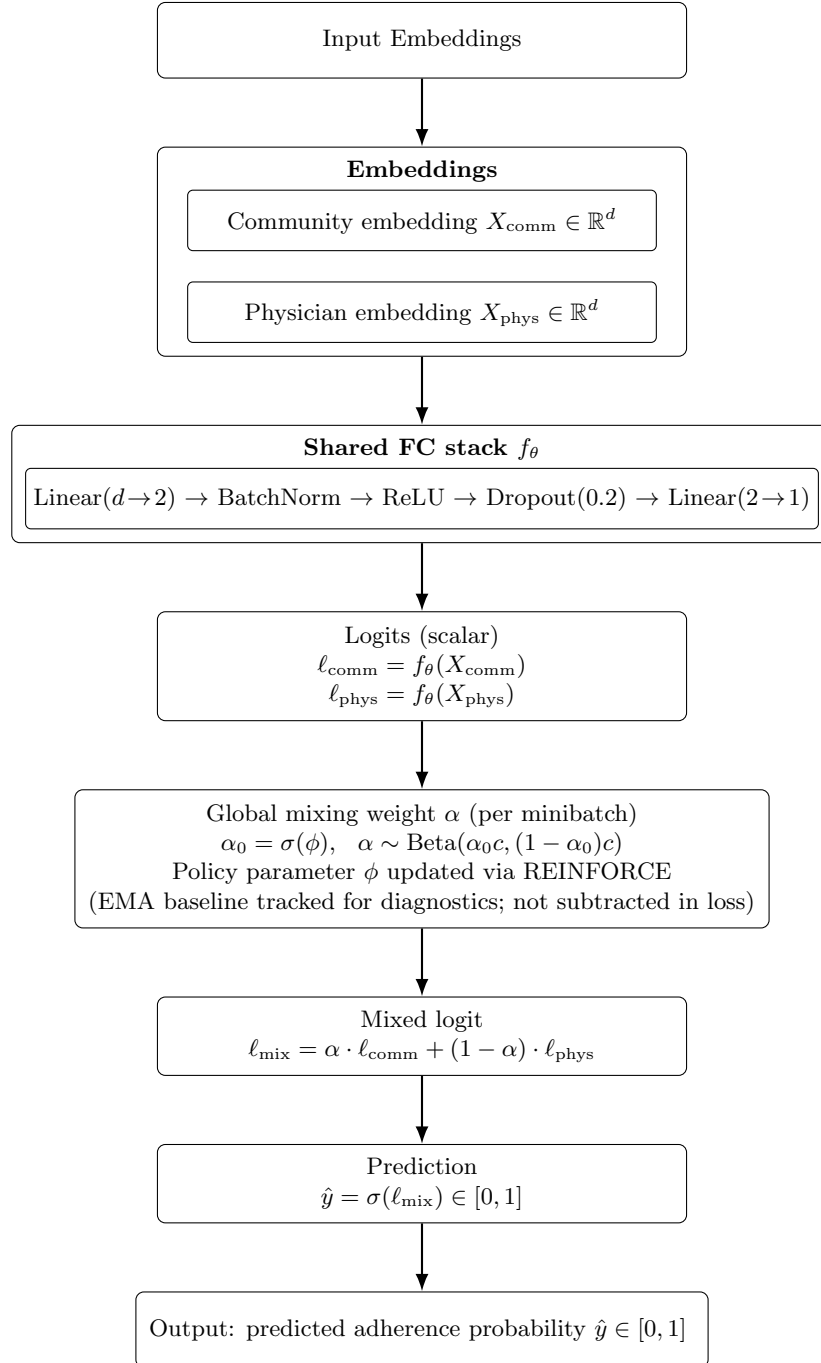

Supplement: S3 File — (PDF) [file pdig.0001294.s003.pdf]
