# Supplementary material for: Participatory-informed preference optimization (PiPrO): A reinforcement learning simulation study
Source: PLOS Digit Health. 2026 Mar 19;5(3):e0001294. doi: 10.1371/journal.pdig.0001294 (PMC13001916; doi:10.1371/journal.pdig.0001294)

S4a. Summary Statistics

	N	Mean	SD	Min	Median	Max
id	10000	5000.5	2886.8957	1	5000.5	10000
efficacy	10000	0.7012	0.1392	0.14	0.71	0.99
toxicity	10000	0.1347	0.153	0	0.09	0.94
patient_urgency_medication	10000	0.4977	0.1494	0.08	0.5	0.93
patient_urgency_lifestyle	10000	0.5023	0.1801	0	0.5	1
provider_urgency_medication	10000	0.5006	0.1483	0	0.5	0.99
provider_urgency_lifestyle	10000	0.5006	0.1778	0	0.5	1
true_adherence_probability	10000	0.377	0.1262	0.05	0.39	0.77
u_med	10000	0.2524	0.1489	0	0.25	0.88
u_life	10000	0.4067	0.1681	0	0.41	0.97
y_star	10000	0.2785	0.4483	0	0	1
patient_pref	10000	0.3438	0.475	0	0	1
provider_pref	10000	0.8236	0.3812	0	1	1
trust_parameters.community_trust	10000	0.7779	0.1323	0.1826	0.7992	0.9969
trust_parameters.physician_trust	10000	0.5002	0.1898	0.0094	0.4993	0.9782
trust_parameters.side_effect_disclosure	10000	0.5001	0.0759	0.3038	0.4997	0.6913
trust_parameters.adherence_honesty	10000	0.5751	0.0664	0.4033	0.5747	0.7424
trust_parameters.financial_disclosure	10000	0.7334	0.0397	0.5548	0.7398	0.7991
trust_parameters.symptom_exaggeration	10000	0.199	0.115	0	0.2005	0.3999

S4b. Pearson Correlation Matrix – Numeric Features

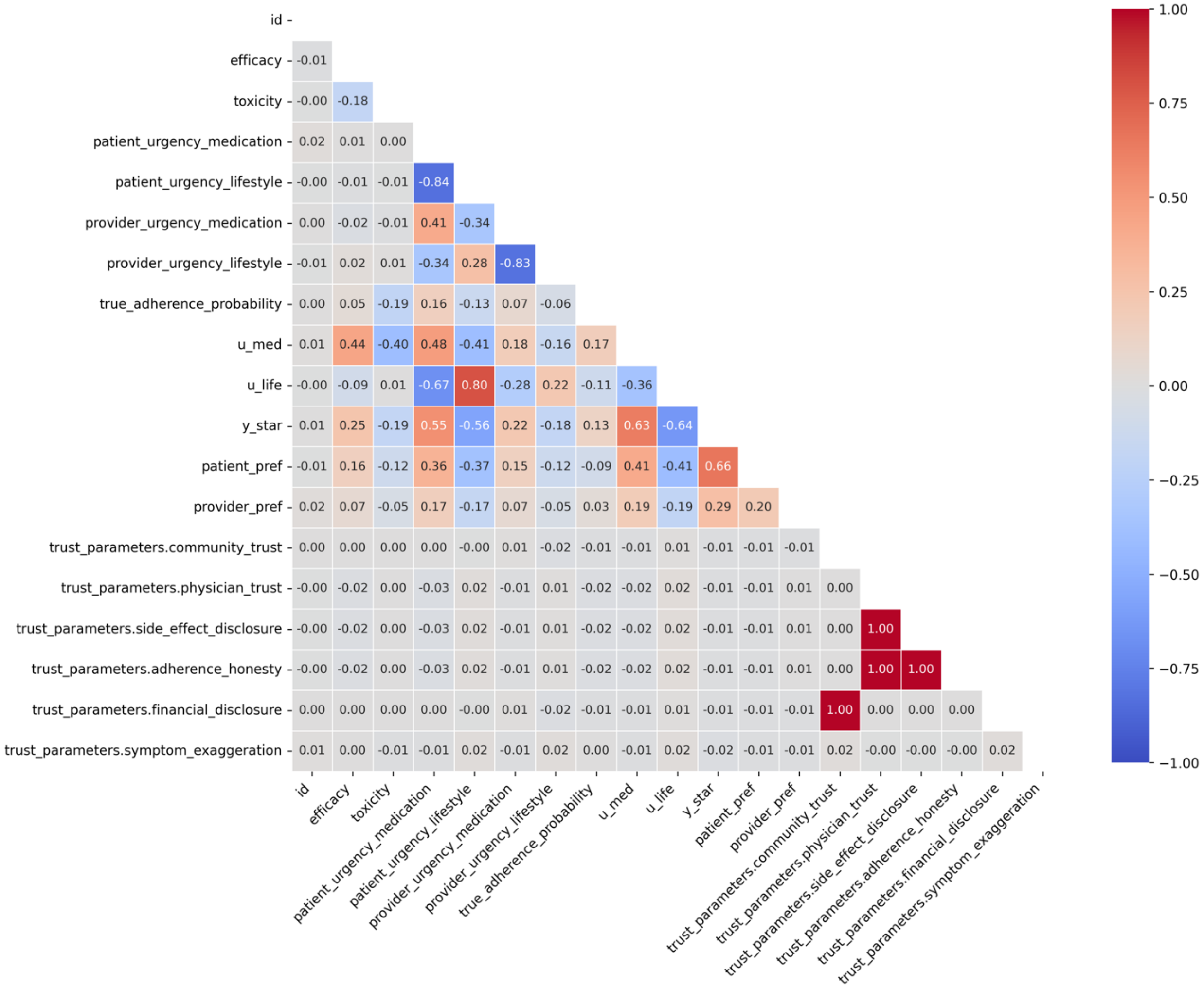

Supplement: S4 File — (PDF) [file pdig.0001294.s004.pdf]
